# Effect of chronic exercise on myocardial electrophysiological heterogeneity and stability. Role of intrinsic cholinergic neurons: A study in the isolated rabbit heart

**DOI:** 10.1371/journal.pone.0209085

**Published:** 2018-12-18

**Authors:** Luis Such-Miquel, Laia Brines, Antonio M. Alberola, Manuel Zarzoso, Francisco J. Chorro, Juan Guerrero, Germán Parra, Nathalia Gallego, Carlos Soler, Irene Del Canto, Antonio Guill, Luis Such

**Affiliations:** 1 Department of Physiotherapy, Universitat de València, Valencia, Spain; 2 Health Research Institute (INCLIVA), Valencia, Spain; 3 Department of Physiology, Universitat de València, Valencia, Spain; 4 Department of Medicine, Universitat de València, Valencia, Spain; 5 Department of Electronic Engineering, Universitat de València, Valencia, Spain; 6 ITACA, Universitat Politècnica de València, Valencia, Spain; Universitatsklinikum Wurzburg, GERMANY

## Abstract

A study has been made of the effect of chronic exercise on myocardial electrophysiological heterogeneity and stability, as well as of the role of cholinergic neurons in these changes. Determinations in hearts from untrained and trained rabbits on a treadmill were performed. The hearts were isolated and perfused. A pacing electrode and a recording multielectrode were located in the left ventricle. The parameters determined during induced VF, before and after atropine (1μM), were: fibrillatory cycle length (VV), ventricular functional refractory period (FRPVF), normalized energy (NE) of the fibrillatory signal and its coefficient of variation (CV), and electrical ventricular activation complexity, as an approach to myocardial heterogeneity and stability. The VV interval was longer in the trained group than in the control group both prior to atropine (78±10 vs. 68±10 ms) and after atropine (76±8 vs. 67±10 ms). Likewise, FRPVF was longer in the trained group than in the control group both prior to and after atropine (53±8 vs. 42±7 ms and 50±6 vs. 40±6 ms, respectively), and atropine did not modify FRPVF. The CV of FRPVF was lower in the trained group than in the control group prior to atropine (12.5±1.5% vs. 15.1±3.8%) and, decreased after atropine (15.1±3.8% vs. 12.2±2.4%) in the control group. The trained group showed higher NE values before (0.40±0.04 vs. 0.36±0.05) and after atropine (0.37±0.04 vs. 0.34±0.06; p = 0.08). Training decreased the CV of NE both before (23.3±2% vs. 25.2±4%; p = 0.08) and after parasympathetic blockade (22.6±1% vs. 26.1±5%). Cholinergic blockade did not modify these parameters within the control and trained groups. Activation complexity was lower in the trained than in the control animals before atropine (34±8 vs. 41±5), and increased after atropine in the control group (41±5 vs. 48±9, respectively). Thus, training decreases the intrinsic heterogeneity of the myocardium, increases electrophysiological stability, and prevents some modifications due to muscarinic block.

## Introduction

It has been reported that aerobic physical exercise can protect against cardiac sudden death, which in most cases is produced by ventricular fibrillation (VF) [1,2], and experimental studies have evidenced that exercise leads to reduced VF [3]. However the exact underlying cardiovascular protective mechanisms are not fully known [4,5]. In this regard, research has been conducted to elucidate whether electrophysiological modifications produced by training could represent a possible protective mechanism. Reports have shown that training increases ventricular electrical stability [6], decreases electrophysiological heterogeneity in *in vivo* canine models [7], and increases the VF threshold during coronary occlusion in isolated rat hearts [8]. We have also reported that physical training increases ventricular refractoriness in trained rabbits [9,10]. Additional electrophysiological modifications produced by training have been investigated [11]. However, the effect of chronic physical exercise upon the intrinsic electrophysiological heterogeneity of the myocardium has been less studied in *in vitro* models. Myocardial heterogeneity is a well known proarrhythmic factor [12].

On the other hand, the cardiac nervous system, which has been suggested to act as the final coordinator of regional cardiac indices [13], can generate spontaneous activity [14] independently of central neuronal inputs [15], and in the absence of vagal impulse activity, acetylcholine is released from cholinergic neurons in mammals in a non-quantal manner [16]. Although many studies have focused on the participation of the extrinsic nervous system in the electrophysiological modifications caused by training, there is less knowledge about the direct influence of cholinergic myocardial neurons upon intrinsic electrophysiological heterogeneity and stability modifications due to physical training. Thus, in the present study conducted in the isolated, perfused and fibrillating rabbit heart, we have investigated the effects of chronic physical exercise upon ventricular electrophysiological heterogeneity and stability through the analysis of fundamental electrophysiological parameters and their ventricular dispersion, together with the complexity of myocardial activation during VF (which is also an associated parameter). Likewise, we have investigated the participation of myocardial cholinergic neurons in the modifications of these parameters. We hypothesize that myocardial physical training decreases electrophysiological heterogeneity and increases myocardial stability, and furthermore that intrinsic myocardial cholinergic neurons are implicated in these changes.

## Material and methods

### Animals and study design

Housing conditions and experimental procedures were in accordance with the European Convention for the Protection of Vertebrate Animals used for Experimental and other Scientific Purposes (Council of Europe No. 123, Strasbourg 1985) and European Union Directive 2010/63 on the protection of animals used for scientific purposes, and were approved by the Institutional Animal Care and Use Committee of the University of Valencia as promulgated by Spanish legislation (RD 53/2013). Two groups of male New Zealand rabbits, nine weeks old and weighing 2,02 ± 0,11 kg, were studied. One group of eleven rabbits was trained on a motor-driven treadmill following a previously described protocol [9], and the other group of ten rabbits (control group) was housed in the animal quarters for the same period. Briefly, in the trained group, rabbits underwent a running program after four days of becoming familiarized with the treadmills, and they ran 5 days a week for 6 weeks at a speed of 0.33 m/s. Each of the training sessions was divided into 6 periods consisting of four minutes of running and one minute of rest between periods.

### Preparation and perfusion

Following heparinization and anesthesia (ketamine 10 mg/kg i.v.), the animals were euthanized, and after a midsternal thoracotomy, the heart was quickly removed and immersed in cold Tyrode solution for further preparation. The aorta was cannulated and connected to a Langendorff system to provide the heart with the warmed and oxygenated Tyrode solution containing (millimolar): NaCl 130, KCl 4.7, CaCl_2_ 2.2, MgCl_2_ 0.6, NaH_2_PO_4_ 1.2, NaHCO_3_ 24.2, and glucose 12. The pH was maintained at 7.4 by equilibration with a mixture of 95% O_2_ and 5% CO_2_. Tyrode temperature was constant throughout the experiment (37° C), and the perfusion pressure was kept at 60 mmHg.

One bipolar surface electrode (Teflon-coated silver wire, with an inter-electrode distance of 1 mm) was positioned on the right atrium for recording, and another on the left ventricle for pacing. Ventricular recordings were made by means of a plaque with 240 unipolar stainless steel electrodes (0.125 mm electrode diameter and 1 mm inter-electrode distance) positioned on the epicardial surface of the posterior wall of the left ventricle. The indifferent electrode was a silver plaque located over the cannulated aorta. Recordings were obtained with a cardiac electrical activity mapping system (MAPTECH, Waalre, The Netherlands). The electrograms were amplified with a gain of 100–300, broad-band (1–400 Hz) filtered, and multiplexed. The sampling rate in each channel was 1 kHz.

Ventricular fibrillation was induced by pacing at increasing frequencies from 4 to 20 Hz, and coronary perfusion was maintained during the arrhythmia. Ventricular fibrillation was triggered when the stimulation frequency reached 816±52 beats per minute in the control group and 773±86 beats per minute in the trained group, the difference not being statistically significant. Muscarinic receptor blockade with atropine did not produce modifications in the stimulation frequency triggering VF in either group. Electrical stimuli for inducing VF (2 ms duration and three times the diastolic threshold) were delivered by a Grass S-88 stimulator (Grass Instruments, Inc., Quincy, MA, USA) fitted to two stimuli isolation units. Data analysis was carried out involving a data block of 4096 points. Data processing was performed with Matlab software on a Hewlett-Packard platform (Hewlett-Packard Co., Palo Alto, CA, USA).

### Measurements and calculations

**Fig 1:**
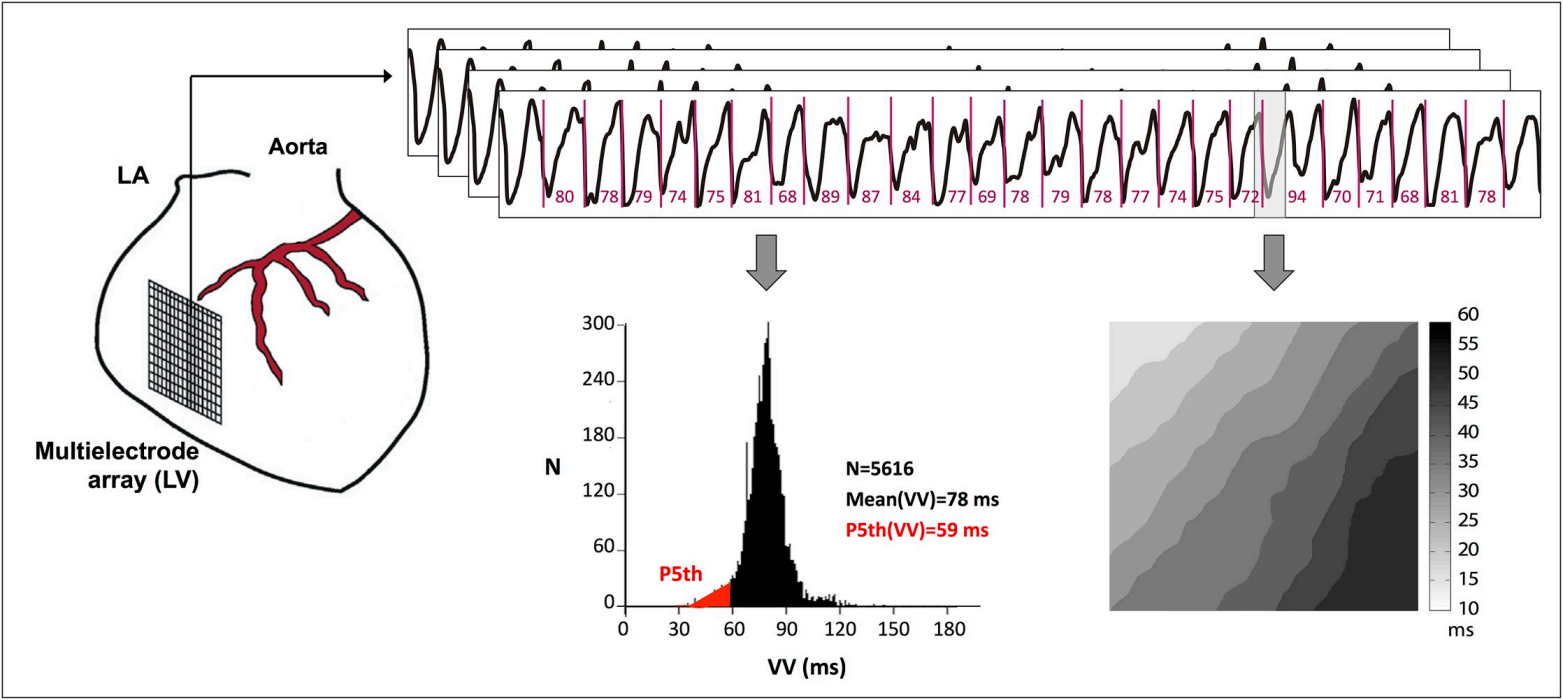
Schematic representation of the methodology used for VF analysis. Location of the multielectrode array on the left ventricle (left). Sample of activation times and VV intervals determined for some electrodes during 2-second VF recordings (top right). The VV intervals histogram was constructed from VF cycles, and the P5th and mean were calculated (bottom left). The activation map during VF was constructed from activation times in a window of 50 ms width (bottom right). VF, ventricular fibrillation; mean (VV), mean of the consecutive activation intervals during ventricular fibrillation; P5th (VV), fifth percentile of the consecutive activation intervals during ventricular fibrillation; N, number of marks and/or intervals.

**Fig 2:**
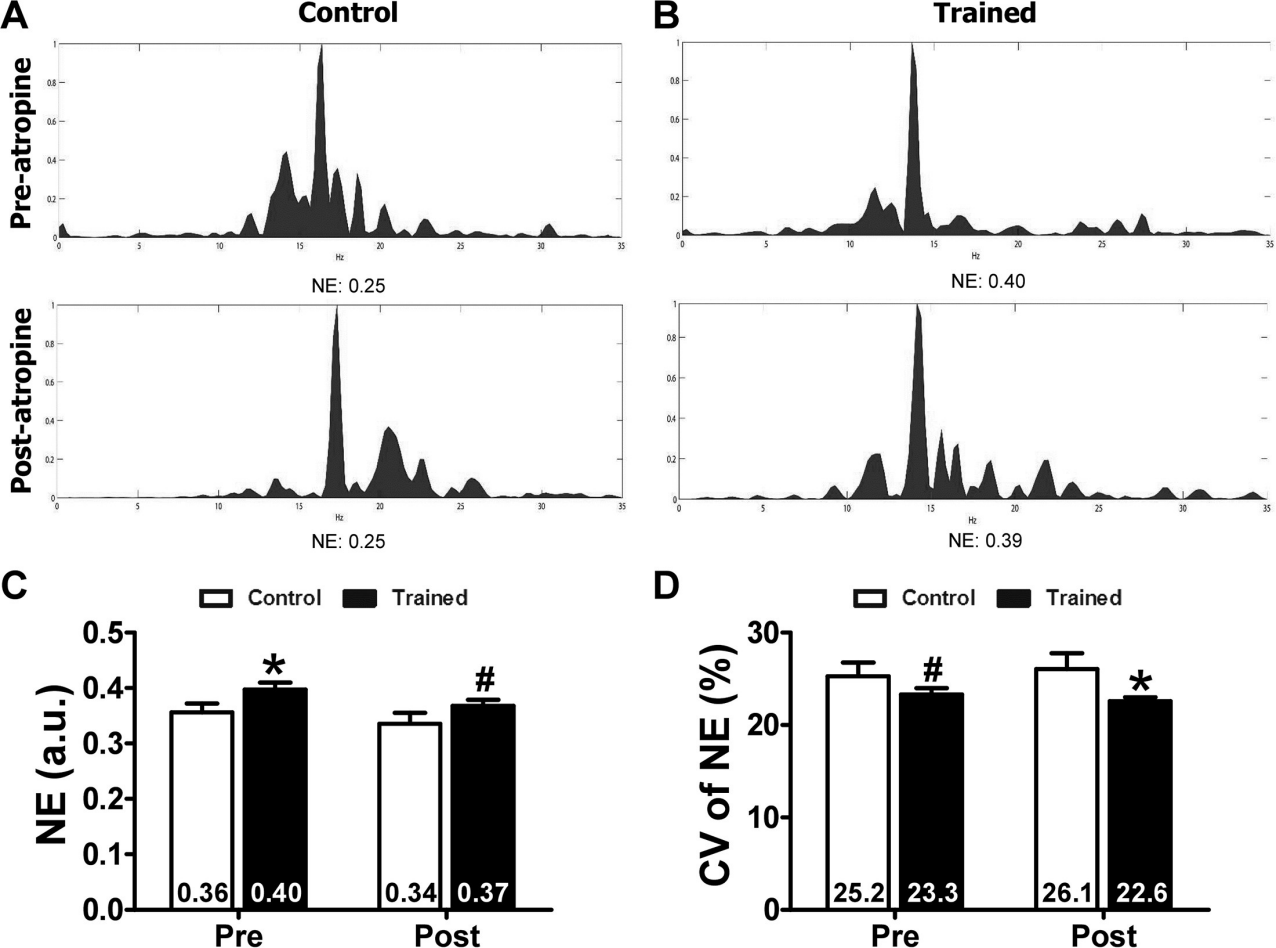
Normalized energy (NE) in control and trained animals before and after parasympathetic blockade. (A) and (B) depict representative traces of the power spectrum density in control (n = 10) and trained (n = 11) animals. NE was higher in the trained group (C), and heterogeneity was lower in the trained group than in control group (D). Cholinergic blockade with atropine did not modify these training-induced electrophysiological modifications (C) and (D). *p<0.05; #p = 0.08; error bars: SEM.

The study parameters were: a) Mean fibrillatory cycle length (VV). Activation times in each electrode were determined by identifying the moment of maximum negative slope of the electrograms. Accordingly, marks were placed on each slope corresponding to the activation waves of the electrograms, thus allowing identification of the VV intervals. The minimal threshold for dV/dt to be considered as a local deflection was a percentage (20%) of the maximal negative slope in each channel. In this way, the fibrillation interval (VV) histograms and the mean of the consecutive VV intervals were determined (Fig 1); b) Ventricular functional refractory period (FRPVF) as the 5th percentile of the VV intervals histogram, constructed from 4500 to 5000 consecutive VF cycles (detailed explanation in Fig 1); c) The coefficient of variation (CV) of FRPVF in each of the 240 unipolar electrodes; d) Normalized energy (NE) of the fibrillatory signal in each of the electrodes, defined as the spectral energy in a window centered on the dominant frequency (DF ± 1 Hz) and normalized by the spectral energy in the band of interest (5–35 Hz). This latter parameter was obtained by processing the recordings in consecutive segments of 4 s each (4000 samples). For each segment and channel we obtained the spectrum from the modified Welch periodogram using Hanning’s window and two non-overlapping stretches with a resolution of 0.5 Hz (see Appendix); e) The CV of NE in each of the electrodes; and e) The epicardial complexity of electrical activation of the ventricular myocardium during the arrhythmia. To determine this latter parameter, epicardial activation maps during VF were generated. Activation maps during VF every 100 ms for one second in windows of 50 ms width were constructed. The number of windows analyzed was 11 in one second. Isochrones were drawn, and in each map the wavefronts, reentrant activations, breakthrough patterns, etc., were analyzed (Fig 2). A numerical value, depending on the number of activation processes observed in the analyzed time windows was assigned to each activation map. When only one activation process appeared (e.g., a reentry, a single front, or breakthrough), the assigned value was one. The sum of the numbers indicating the activation complexity detected in each of the analyzed windows was then calculated.

### Experimental series and protocol

Two experimental groups (control and trained) were studied. The experimental protocol in each group was as follows: once the hearts were isolated and perfused (Langendorff system), VF was induced by pacing at increasing frequencies from 4 Hz up to the onset of VF. Coronary perfusion was maintained during the arrhythmia. Once VF was triggered, recordings were obtained for 5 minutes, after which we proceeded to defibrillation of the hearts. At this time an atropine solution was infused through the root of the aorta, reaching 1 μM in the heart, until the end of the experiment. Ventricular fibrillation was again induced and recordings were obtained 5 minutes after onset of the arrhythmia. Perfusion pressure was controlled during the experiment, and the hearts were weighed at the end of the experiments. Measurements of the parameters were determined 5 minutes after VF onset and after atropine administration.

### Data analysis

Two-way analysis of variance (ANOVA) with repeated measures on one factor and an unpaired *t* test were used when appropriate. Values of *p*<0.05 were considered statistically significant.

## Results

### VV intervals

The mean of the VV intervals was longer in the trained group than in the control group prior to atropine administration (78 ± 10 vs. 68 ± 10 ms) and after atropine administration (76 ± 8 vs. 67 ± 10 ms). No significant differences were found before and after atropine administration in any group.

### Functional refractory period during ventricular fibrillation

The FRPVF was longer in the trained group than in the control group prior to atropine administration (53 ± 8 vs. 42 ± 7 ms, respectively). This parameter did not change after atropine versus the pre-atropine values in either the control group (40 ± 6 vs. 42 ± 7 ms, respectively) or in the trained group (53 ± 8 vs. 52 ± 6 ms, respectively).

### Coefficient of variation of FRPVF

The CV of FRPVF was higher in the control group than in the trained group prior to atropine administration (15.1 ± 3.8% vs. 12.5 ± 1.5%), but not after atropine (12.2 ± 2.4% vs. 10.8 ± 2.1%). This parameter decreased after atropine in the control group (15.1±3.8% vs. 12.2±2.4%) but not in the trained group (12.5 ± 1.5% vs. 10.8 ± 2.1%).

### Normalized energy of the fibrillatory signal

Hearts from trained animals showed higher NE values before (0.40±0.04 vs. 0.36±0.05; p<0.05) and after atropine administration (0.37±0.04 vs. 0.34±0.06; p = 0.08) (Fig 1C). Physical training decreased the heterogeneity of this parameter as shown by the CV of NE, both before (23.3±2 vs. 25.2± 4%; p = 0.08) and after parasympathetic blockade (22.6±1 vs. 26.1 ± 5%; p<0.05) (Fig 1D). On the other hand, cholinergic blockade did not modify these parameters within the control and trained groups.

### Coefficient of variation of NE of the fibrillatory signal

Physical training decreased the heterogeneity of NE of the fibrillatory signal as shown by the CV of NE, both before (23.3±2 vs. 25.2± 4%; p = 0.08) and after parasympathetic blockade (22.6±1 vs. 26.1±5%; p<0.05) (Fig 1D). On the other hand, cholinergic blockade did not modify these parameters within the control and trained groups.

### Activation complexity

**Fig 3:**
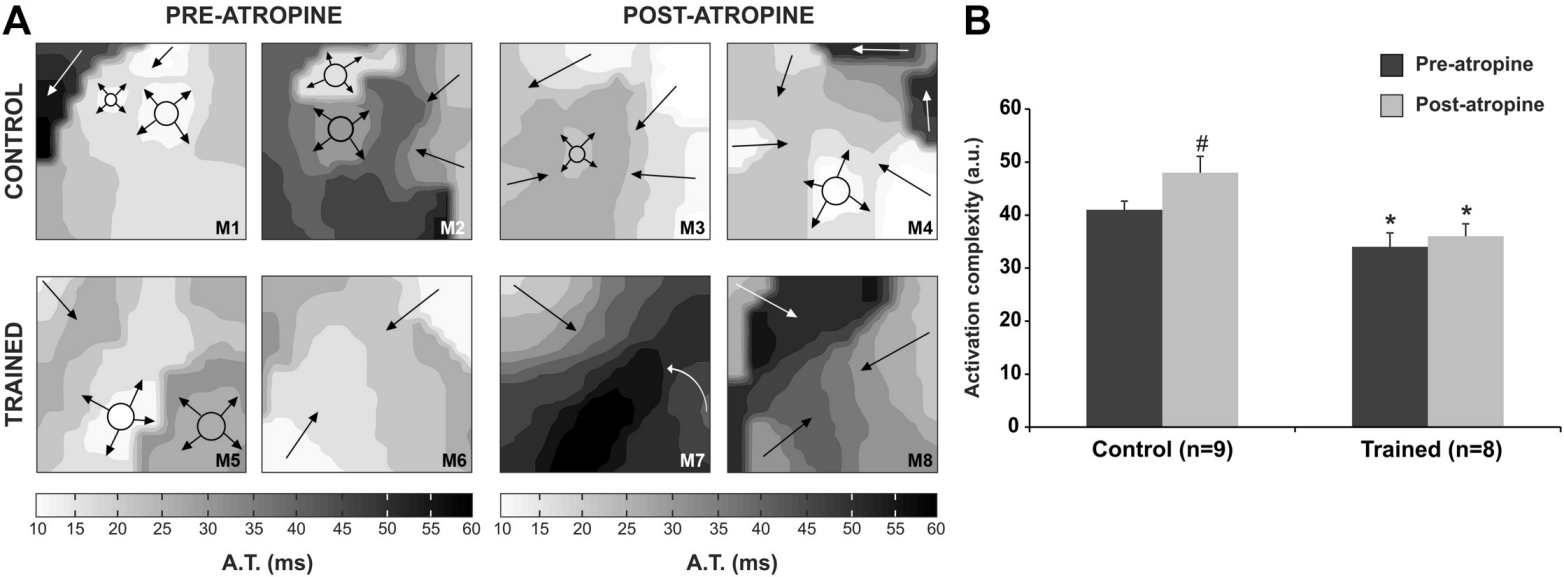
Myocardial activation complexity during ventricular fibrillation. (A) Bidimensional representation of two activation maps obtained in both situations (pre- and post-atropine), in an experiment of the control group and another experiment of the trained group. In each map, the number of activation processes observed determines activation complexity level: in map 2 (M2), arrows represent each one a wavefront and circle with arrows represent breakthrough activation, resulting in a total of 4 activation processes and the assigned activation complexity is 4; map 8 (M8) shows 3 simultaneous wavefronts, and the assigned activation complexity is 3. The more simple activation maps are seen to predominate in trained group before and after atropine infusion. (B) Myocardial activation complexity in control and trained group, before and after atropine administration. *p<0.05 vs. control; #p<0.05 vs. pre-atropine. A.T., activation time; M, map.

In terms of the characteristics of the activation maps during VF, the activation complexity was greater in the control group than in the trained animals prior to atropine (41 ± 5 vs. 34 ± 8) and after atropine (48±9 vs. 36±7). This parameter increased in the control group after atropine versus the prior to atropine values (41 ± 5 vs. 48 ± 9 ms, respectively). No differences were observed in the trained group (36 ± 7 vs. 34 ± 8 ms, respectively). See Fig 3.

### Heart and body weight of rabbits and heart–to–body weight ratio

No differences in cardiac mass were found between the control and trained groups (14.4 ± 1.9 g; n = 11 vs. 14.1 ± 2.2 g; n = 12). Likewise, there were no differences in the weight of the rabbits (3.63 ± 0.25 kg; n = 11 vs. 3.54 ± 0.41 kg; n = 12) or in the heart-to-body weight ratio (0.0037 ± 0.0004; n = 11 vs. 0.0040 ± 0.0003; n = 12) on comparing the control group with the trained group.

## Discussion

An isolated and fibrillating rabbit heart model has been used to study the effects of chronic physical exercise upon the ventricular myocardium functional refractory period, normalized energy of the fibrillatory signal, and ventricular dispersion of these parameters as an index of myocardial heterogeneity. We have also determined the frequency of activation during ventricular fibrillation, analyzed in the time domain, and ventricular activation complexity during ventricular fibrillation these parameters being indexes of refractoriness, electrical stability and heterogeneity, respectively. The study was made before and after atropine administration in order to investigate the contribution of myocardial cholinergic neurons to the mentioned parameters and their modification through chronic exercise, which remain unknown to date. This experimental study has been performed using the rabbit, which is an ideal model for examining the effects of aerobic physical training. Indeed, it has been reported that by using the proper intensity, duration, and frequency of exercise, the rabbit achieves a documented cardiovascular training effect rather easily [17]. We have previously reported that using the training protocol of the present study, the *in vivo* heart rate was lower in the trained animals than in the non-trained animals. Moreover, sinus cycle length analyzed in the isolated heart, likewise as a cardiovascular parameter of training, was longer in the trained rabbits than in the controls. Not only the cardiovascular parameters evidenced the effect of the applied protocol on a treadmill, but also the biochemical parameters HS60 and iNOS were modified [9]. Since we have determined these parameters during VF (without interrupting perfusion), the rabbit also provide a good model in contrast to other species such as the mouse, because VF in rabbits can be easily induced and sustained [1]. The present model simulates sedentary humans that perform moderate exercise as cardiovascular protection against life-threatening arrhythmias [1].

One of the results in our study is that chronic physical exercise increased the ventricular functional refractory period, and cholinergic blockade did not modify this parameter in trained and non-trained animals. The results also showed a longer mean VV interval in the trained group versus the control group, and no modifications of this interval were observed after cholinergic blockade. The mean VV interval is known to be closely related to refractoriness [18]. The increase in refractoriness due to chronic physical exercise which we have observed has been previously reported using different methodology for assessing this property [9,19,20]. Our results also showed ventricular functional refractory period dispersion to be greater in the control group than in the trained group before atropine administration. Cholinergic blockade did not modify the CV of FRPVF after atropine in the trained group, though a slight but significant decrease was observed in the control group. The determination of the ventricular functional refractory period constructed from multiple consecutive VF cycles from unipolar left ventricle electrograms offers several advantages [20], allowing estimation of the refractory period at over 200 different points of the myocardium, and not only at the site of pacing, as occurs when the extrastimulus test is used. Moreover, by using the mentioned methodology we can also estimate myocardial heterogeneity, analyzing refractory period dispersion through the ventricular myocardium.

In a way similar to the dispersion of refractoriness, the greater NE values and lower CV of the NE found in the trained animals also reinforces the idea that training decreases myocardial electrophysiological heterogeneity. As is known, NE informs about the spectral concentration of the fibrillatory signal. Indeed, a perfectly periodic signal will have all its power spectrum concentrated in the dominant frequency and its harmonics, while an irregular signal will have a lower percentage of the spectrum area under the dominant frequency. Thus, the calculation of this percentage provides information about the organization and regularity of the VF signals, and therefore about electrophysiological heterogeneity, which in turn is associated with the facility of establishing and/or ending VF. The absence of changes in NE and its CV, after atropine administration, also allows us to conclude that cholinergic neurons do not play a direct role in relation to these properties.

Our results concerning ventricular refractoriness as well as the VV interval should be interpreted as a protective mechanism against reentrant arrhythmias. It is well known that myocardial refractoriness is a property, along with conduction velocity, that determines the wavelength of the myocardial activation process, which in turn is a factor closely related to the appearance and stability of reentrant arrhythmias over time [21]. As noted above, cholinergic blockade does not modify refractoriness in trained or non-trained animals, suggesting that myocardial cholinergic neurons do not have a direct effect upon this property under sedentary and trained conditions. The lower values of the parameters of electrophysiological heterogeneity produced by physical training, observed in the present study, and as has been discussed for refractoriness, indicate that endurance physical training could exert an antiarrhythmic effect at least in part by decreasing myocardial electrophysiological heterogeneity. Indeed, the importance of heterogeneity and the refractory period in the induction of cardiac fibrillation has been well established [12]. Thus, if the changes in refractoriness and heterogeneity could participate as an additional basic mechanism in which aerobic exercise exerts a protective effect against reentrant arrhythmias such as VF, which can produce cardiac sudden death [1], then cholinergic neurons do not seem to play a direct role in the electrophysiological adaptations found in the present study.

Regarding the slight decrease in refractoriness dispersion on ventricular myocardium after cholinergic blockade, observed in the control group, although we have not investigated the mechanisms capable of explaining this effect, some considerations should be taken into account. The data accumulated in the last few years give an account of the complexity of intracardiac neurons in modulating heart function. It has been reported that intrinsic cardiac neurons generate spontaneous activity [14] and can function independently of central neuronal inputs [15]. Moreover, recent reports show that in the absence of vagal impulse activity, acetylcholine is released from cholinergic neurons in mammals [16]. In a way similar to the effects exerted by acetylcholine on the atria, shortening the atrial refractory period and increasing its dispersion [22], we speculate that acetylcholine release in the isolated heart ventricle and thus not under central nervous system control, would also increase the dispersion of ventricular refractory periods. This would explain the decrease in ventricular heterogeneity in terms of functional refractory period dispersion during VF after cholinergic blockade.

With respect to electrical activation during VF, we observed lesser electrical activation complexity during VF in the trained group than in the control group. Cholinergic blockade did not modify this parameter in the trained group, though it increased after atropine in the control group. This result should be interpreted as a beneficial effect. Indeed, a decrease in activation complexity implies an increase in myocardial electrical stability, and thus represents a protective effect against the generation of reentrant arrhythmias. The mechanisms by which cholinergic blockade increases the complexity of myocardial activation during VF in the control group have not been investigated in the present study. Although it seems paradoxical that cholinergic blockade decreased electrophysiological heterogeneity in the control group despite an increase in the complexity of activation, no such paradox actually exists. Indeed, it has been reported that drugs like flecainide, sotalol and verapamil, with different electrophysiological actions (i.e., upon refractoriness and the spectral characteristics of ventricular fibrillation), produce the same effect upon the complexity of ventricular activation [18]. It has also been reported that ventricular stretching and verapamil administration increase the dominant frequency of ventricular fibrillation, and consequently the coefficient of variation of the VF dominant frequency [23]. However, ventricular stretch increased the complexity of the myocardial activation during arrhythmia, while verapamil decreased it [18].

On the basis of the results obtained, some considerations should be taken in account. It has been reported that intracardiac neurons can operate with some degree of independence from extrinsic neuronal inputs [24], and that spontaneous activity can occur if the ganglia have been acutely decentralized [25]. This could explain why myocardial cholinergic neurons seem to exert some cholinergic control of electrophysiological heterogeneity as well as of electrical activation complexity during VF, even though these neurons do not seem to influence myocardial refractoriness. Our results suggest the need for further research to explain the mechanisms by which cholinergic neurons directly modify some electrophysiological parameters and also explain the apparent paradox of the changes in these parameters.

It seems especially remarkable that unlike in the control group, the trained rabbits exhibited no modifications in the heterogeneity and complexity of the activation produced by cholinergic blockade. These results lead us to postulate that training seems to reduce sensitivity to cholinergic muscarinic blockade, making the ventricle less susceptible to the direct influence of intrinsic cholinergic neurons upon the electrophysiological parameters investigated. The absence of effects of cholinergic blockade upon the parameters analyzed in fibrillatory rhythm in the trained group and on some parameters in the control group seems to be closely related to the results referred to heart rate and other parameters obtained in sinus rhythm, previously reported by our group [10].

Despite the results obtained in the present study, we must emphasize the interest which the use of M2R-knock-out-rabbits would have in investigating not only the electrophysiological parameters analyzed in this study but also other parameters previously analyzed using our experimental model. This, together with studies based on cholinergic stimulation and/or beta-adrenergic blockade, would make it possible to clarify and probably reinforce our findings referred to the participation of cholinergic neurons in the electrophysiological modifications produced by low intensity training in a sedentary animal such as the laboratory rabbit. We recommend the conduction of such studies in rabbits and not in other species, due to the abovementioned reasons. On the other hand, the effects of physical exercise upon the activity of the sinus node and the underlying mechanisms have been investigated and attributed to the downregulation of hyperpolarization-activated cyclic nucleotide-gated (HCN), Na+, Ca2+ and K+ channel mRNA, and a significant correlation between heart rate and HCN4 mRNA expression has been described as well [26]. Nevertheless, although some of the mentioned components, such as the HCN channels, are present in the ventricular myocardium [27, 28], as far as we know, no information is available about their influence upon the effects induced by exercise training on ventricular electrophysiology. Accordingly, some of these mechanisms should be investigated in the ventricles in order to clarify and obtain basic knowledge about the effects of chronic physical exercise upon the myocardial electrophysiological properties related to establishment of the life-threatening arrhythmias, which we have investigated in the present study.

In conclusion, training by means of a physical exercise protocol in rabbits has produced an increase in ventricular refractoriness, a decrease in ventricular electrophysiological heterogeneity, and a reduction of the complexity of electrical activation during ventricular fibrillation in the isolated and perfused heart. Training seems to reduce sensitivity to cholinergic muscarinic blockade, which does not alter the intrinsic refractoriness, heterogeneity and stability modifications produced by training. The results obtained in the present study raise the need to pursue this research in order to elucidate the mechanisms underlying the observed modifications through both training and the cholinergic blockade S1 Appendix.

## Appendix

For each segment and channel, we obtained the power spectrum by means of the Welch [29] periodogram, which uses the averaging of modified periodograms. In sum, the process is as follows:

1. Divide the data segment, x, into sections that may overlap. In this case, it has been divided into two non-overlapping sections, t1 and t2:
  $$\begin{array}{l} x(n),\;n=1,\ldots ,L\\ x_{t1}(i),\;i=1,\ldots ,L/2\\ x_{t2}(j),j=L/2+1,\ldots ,L \end{array}$$
  L = number of samples.
2. A window is applied to each signal section, resulting in a modified periodogram. Hanning’s window is used, defined by:
  $$\begin{array}{c} w(n)=\frac{1}{2}\{1-\text{cos}\left[\frac{2\pi n}{L/2}\right]\};n=1,\ldots ,L/2 \end{array}$$
3. The periodogram of each section is calculated:
  $$P_{xx}^{ti}(f)=\frac{1}{L/2}\sum_{n=0}^{L/2-1}x_{ti}(n)w(n)e^{j2\pi fn},\;t_{i}=1,2$$
4. The power density spectrum of each segment of the record is given by:
  $$P_{xx}(f)=\frac{1}{2}\sum_{i=1}^{2}P_{xx}^{ti}(f)$$
5. The spectral energy is calculated in the FD ± 1 Hz band, and in the band of interest (5–35 Hz) by integrating the power spectrum in these bands.
6. Finally, we obtain the normalized energy (NE) as the ratio between the spectral energy in the FD ± 1 Hz band and the energy of the band of interest.

## Supporting information

S1 Appendix S1_Minimal_Data_Set.xlsx. Data obtained to calculate each parameter.
(XLSX)
